# ESPO AND BEHAVIORAL AND SOCIAL SCIENCES SECTION SYMPOSIUM: DEFYING THE ODDS: HOW MINORITIZED OLDER ADULTS USE PSYCHOSOCIAL AND ENVIRONMENTAL FORTITUDE TO ENHANCE AGING HEALTH

**DOI:** 10.1093/geroni/igae098.0619

**Published:** 2024-12-31

**Authors:** E-Shien Chang, Monica Walters, Laura Zahodne

**Affiliations:** Chair; Co-Chair; Discussant

## Abstract

Minoritized older adults report worse health outcomes compared to their non-Hispanic White counterparts. However, existing research often focuses on the role of risk factors rather than sources of resilience among minoritized older adults. In this symposium, we will highlight ways in which racially and ethnically minoritized older adults use different facets of fortitude to ameliorate disparities and promote resilience. Our first two presentation will examine psychosocial fortitude in relation to cognitive health. Dr. Alexandra Clark will examine how belonging to a resilient psychosocial-behavioral phenotype buffers risk for Alzheimer’s disease and how bilingualism protects cognitive health variably among Latinx older adults. Dr. Ruijia Chen will address racial/ethnic disparities in Alzheimer’s disease and related dementias by focusing on the interplay of life course stressors, resilience factors, and sleep health across the life span. Our last two presentations will examine environmental factors in relation to cognitive and physical health. Dr. Talha Ali will provide insights on resilience mechanisms by exploring the moderating role of individual-level race/ethnicity in the relationship between structural racism and increased risk for subjective cognitive decline. Dr. Kyle Moored will explore ways in which neighborhood walkability buffers the negative impact of neighborhood socioeconomic disadvantage on step activity among Black older persons. Dr. Laura Zahodne, as discussant, will provide summative comments identifying synergies across presentations and promoting strategies for advancing the field of minority aging research.

